# Electronic properties of core-shell nanowire resonant tunneling diodes

**DOI:** 10.1186/1556-276X-9-509

**Published:** 2014-09-18

**Authors:** Matthew Zervos

**Affiliations:** 1 Nanostructured Materials and Devices Laboratory, Nanotechnology Research Center, University of Cyprus, 1678 Nicosia, Cyprus; 2 Department of Mechanical and Manufacturing Engineering, School Of Engineering, University of Cyprus, PO Box 20 537, Nicosia 1678, Cyprus

**Keywords:** III-V, Core-shell, Nanowires, Electronic properties

## Abstract

The electronic sub-band structure of InAs/InP/InAs/InP/InAs core-shell nanowire resonant tunneling diodes has been investigated in the effective mass approximation by varying the core radius and the thickness of the InP barriers and InAs shells. A top-hat, double-barrier potential profile and optimal energy configuration are obtained for core radii and surface shells >10 nm, InAs middle shells <10 nm, and 5 nm InP barriers. In this case, two sub-bands exist above the Fermi level in the InAs middle shell which belongs to the *m* = 0 and *m* = 1 ladder of states that have similar wave functions and energies. On the other hand, the lowest *m* = 0 sub-band in the core falls below the Fermi level but the *m* = 1 states do not contribute to the current transport since they reside energetically well above the Fermi level. We compare the case of GaAs/AlGaAs/GaAs/AlGaAs/GaAs which may conduct current with smaller applied voltages due to the larger effective mass of electrons in GaAs and discuss the need for doping.

## Background

**Figure 1:**
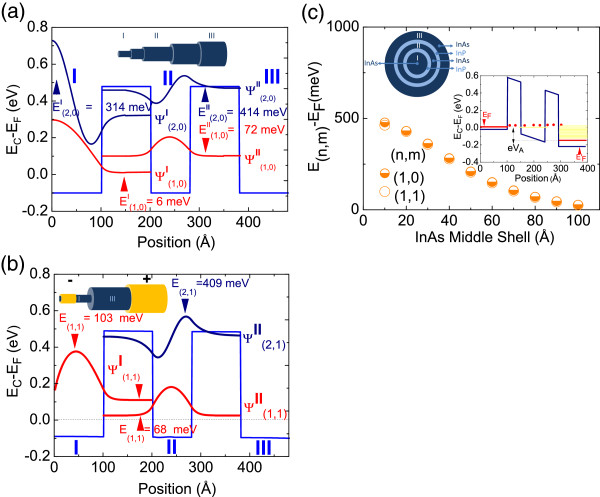
**CB profile, sub-band energies and wave functions and variation of energy sub-band versus thickness. (a)** CB profile, sub-band energies and wave functions for *m* = 0 in a 10-nm InAs/10 nm InP/8 nm InAs/10 nm InP/10 nm InAs core-shell NW and **(b)** for *m* = 1 in the same core-shell NW of **(a)**. **(c)** Variation of the lowest *m* = 0, *n* = 1 and *m* = 1, *n* = 1 sub-band energies versus thickness of the middle InAs shell shown in **(a)** or **(b)**.

Semiconductor nanowires (NWs) with a changing composition in the axial or radial direction such as axial InAs/InP/InAs/InP/InAs NW resonant tunneling diodes (RTDs) [1] and InGaAs/InP/InAlAs/InGaAs core-shell NW field effect transistors (FETs) [2] are attractive for the fabrication of emerging devices in view of the ongoing downscaling of Si integrated circuits (ICs). NW RTDs are also interesting from a fundamental point of view and a detailed investigation of the current transport properties of a 2-μm long InAs/5 nm InP/15 nm InAs/5 nm InP/InAs axial NW RTD was first carried out by Björk et al*.*[1]. The InAs/InP/InAs/InP/InAs NW was grown along the [111] direction, had a radius of 20 nm, and exhibited a sharp peak in the current–voltage characteristic at an applied voltage 80 mV at 4.2 K having a full-width half-maximum of 5 mV attributed to resonant tunneling of carriers through the double barriers. The current transport properties and electronic sub-band structure of these axial InAs/InP/InAs/InP/InAs NW RTDs were calculated previously via the self-consistent solution of the Poisson-Schrödinger equations in the effective mass approximation which showed that resonance occurs at *V*_A_ = 88 mV in good agreement with experiment [3,4]. However, to date, there are no experimental or theoretical investigations of InAs/InP/InAs/InP/InAs core-shell NW RTDs despite the fact that these are expected to have a larger current-carrying capability compared to their axial counterparts. This is due to the fact that electrons tunnel, one at a time, via discrete states confined in the middle InAs cylindrical quantum dot in axial InAs/InP/InAs/InP/InAs NW RTDs. In contrast, many electrons can tunnel at the same time via one-dimensional sub-band states confined in the InAs middle shell of a InAs/InP/InAs/InP/InAs core-shell NW RTD which in turn is expected to yield a larger current-carrying capability. A typical InAs/InP/InAs/InP/InAs core-shell NW RTD including two ohmic contacts on the core and surface shells is shown as an inset in Figure 1a,b. Here, we should point out that recent efforts into the fabrication of high performance InGaAs/InP/InAlAs/InGaAs core-shell NW FETs on Si have demonstrated the feasibility and potential of core-shell NWs for increasing the speed of ICs [2]. Consequently, InAs/InP/InAs/InP/InAs core-shell NW RTDs can be grown in a similar fashion not only for the fabrication of novel nanoscale devices in view of the ongoing downscaling of ICs but also for energy conversion applications despite the fact that they require extra processing compared to their axial counterparts. More specifically, InAs/InP/InAs/InP/InAs core-shell NW RTDs are attractive not only for carrier filtering in high thermoelectric figure of merit devices which must possess a high current-carrying capability but also for the realization of hot carrier solar cells [5].

Here, the electronic properties and sub-band structure of InAs/InP/InAs/InP/InAs core-shell NW RTDs such as the energetic position of the one-dimensional sub-bands, corresponding wave functions, and potential profile in the radial direction have been investigated via the self-consistent solution of the Poisson-Schrödinger (SCPS) equations in cylindrical coordinates and the effective mass approximation taking into account a one-dimensional density of states and by varying the thicknesses of the core radius, barrier, and middle and surface shell thicknesses thereby complementing earlier investigations on axial InAs/InP/InAs/InP/InAs NW RTDs [3,4]. It should be noted that the electronic sub-band structure of axial NW RTDs and core-shell NW FETs have also been calculated using SCPS solvers in the effective mass approximation which is valid since their radii are of the order of many tens of nm's [2-4].

An optimum energy configuration is obtained for InAs/InP/InAs/InP/InAs core-shell NW RTDs with core radii and surface shells >10 nm, InAs middle shell thicknesses of 5 to 10 nm, and 5 nm InP barriers. We find that two sub-bands are energetically located above the Fermi level in the InAs middle shell belonging to the *m* = 0 and *m* = 1 ladder of angular momentum states which have similar wave functions and energies. On the other hand, the lowest *m* = 0 sub-band falls below the Fermi level in the core, but the *m* = 1 states do not play a significant role in the current transport since they reside energetically higher above the Fermi level. We compare the case of GaAs/AlGaAs/GaAs/AlGaAs/GaAs NW RTDs which may conduct current with smaller applied voltages but require doping since the Fermi level is pinned at the middle of the gap giving rise to depletion rather than inversion which occurs at the surface of InAs/InP/InAs/InP/InAs core-shell NW RTDs.

## Methods

Here, we will consider the case of an infinitely long InAs/InP/InAs/InP/InAs core-shell NW RTD in cylindrical coordinates so that the effective mass m^*^ and permittivity *ϵ*_r_ do not depend on − *z* or − *θ*. A schematic diagram of such a core-shell NW RTD is shown as an inset in Figure 1a,b,c. In this case, electrons are free along − *z* but are confined in − *r* and − *θ* and occupy one-dimensional sub-bands in the conduction band (CB) forming a one-dimensional electron gas (1DEG) charge distribution. In order to determine the energetic position of the one-dimensional sub-bands, their occupancy, overall charge distribution, and band bending is necessary to solve the Poisson-Schrödinger equations in − *r* and − *θ* in a self-consistent fashion. In such a self-consistent calculation, Schrödinger's equation is initially solved for a trial potential *V*, and the charge distribution *ρ* is subsequently determined by multiplying the normalized probability density, ∣*ψ*_*k*_∣^2^, by the thermal occupancy of each sub-band with energy *E*_k_ using Fermi-Dirac statistics and summing overall *k* which will be used interchangeably with the integer *n* corresponding to quantization in − *r*. The Poisson equation is solved for this charge distribution in order to find a new potential *V*′, and the process is repeated until convergence is reached. Consequently, we consider first Schrödinger's equation in − *r* and − *θ* which is given by the following:

(1) $$-\frac{\hbar^{2}}{2}\left[\frac{\partial }{\partial r}\frac{1}{m^{*}}\frac{\partial \psi }{\partial r}+\frac{1}{r}\frac{1}{m^{*}}\frac{\partial \psi }{\partial r}+\frac{1}{r^{2}}\frac{\partial }{\partial \theta }\frac{1}{m^{*}}\frac{\partial \psi }{\partial \theta }\right]+\mathrm{V\psi}=\mathrm{E\psi}$$

where *ħ* is Planck's constant divided by 2*π*, m^*^(*r*,*θ*) is the effective mass of the electron, *V*(*r*,*θ*) is the conduction band edge potential, *ψ*(*r*,*θ*) is the wave function, and *E*_*n*_ is the corresponding sub-band energy. We assume that the potential is circularly symmetric and hence, we consider wave functions of the form *ψ*(*r*,*θ*) = *ψ*(*r*)*e*^i*mθ*^ where *m* = 0, ±1, ±2, ±3,… is the angular momentum quantum number corresponding to quantization in − *θ*. The potential *V*(*r*) is related to the electrostatic potential ϕ(*r*) via

(2) $$V\left(r\right)=-\mathrm{e\varphi}\left(r\right)+\Delta {E_{C}}^{i,i+1}\left(r\right)$$

where Δ*E*_*C*_^*i,i+1*^*(r)* is a pseudopotential due to the band offset between materials of different composition in adjacent shells *i* and *i +* 1*.* Finite differences are used to express Schrödinger's equation into a standard matrix eigenvalue problem, i.e.,

(3) $$H_{m}\Psi_{m}=E_{m}\Psi_{m}\text{,}$$

where *H*_m_ is the Hamiltonian matrix for a specific angular momentum number *m*, which is described in detail elsewhere [3]. Each sub-band *E*_*n*,*m*_ is therefore labeled by *n*, *m*. In order to verify the correctness of the matrix setup, the ground state or lowest sub-band energy and wave function were calculated for a GaAs/AlGaAs core-shell NW consisting of a 100 Å GaAs core surrounded by a 100 Å AlGaAs shell, taking into account m^*^_AlGaAs_ = 0.092 m_o_, m^*^_GaAs_ = 0.067 m_o_ and a conduction band discontinuity of Δ*E*_C_ = 0.23 eV at the GaAs/AlGaAs heterointerface, by solving the Schrödinger equation. The step-like, flat-band potential was taken to be zero inside the GaAs core and equal to *E*_C =_ 0.23 eV in the AlGaAs shell. The lowest sub-band energy *E*_*n*=1*,m*=0_ = 24 meV was found to be in excellent agreement with the numerical results of Harrison [6] derived using the shooting method. The dependence of *E*_*n*=1,*m*=0_ as a function of the radius *R* was also checked and found to be in agreement with Harrison [6], e.g., for a 40 Å GaAs/100 Å AlGaAs core-shell NW, *E*_*n*=1,*m*=0_ = 96 meV. In the case of an infinitely long nanowire, the wave functions take the form *ψ*(*r*,*z*,*θ*) = *ψ*(*r*)*e*^*imθ*^*e*^*i*l*z*^ and are normalized according to the following:

(4) $${\left(2\pi \right)}^{2}\int_{0}^{R}r{\left|\Psi \left(r\right)\right|}^{2}\mathrm{dr}=1\text{,}$$

where *R* is the radius of the nanowire. The 1DEG density is given by the expression

(5) $$n_{1\mathrm{DEG}}\left(r\right)=\sum_{k}n_{k}{\left|\Psi_{k}\left(r\right)\right|}^{2}\text{,}$$

where summation runs over the sub-bands and for each *k* the angular momentum quantum number takes on the values *m* = 0, ±1, ±2, ±3,…. Furthermore *n*_*k*_ is the thermal occupancy of the *k*th sub-band which is given by the following:

(6) $$n_{k}=\frac{1}{\pi }\sqrt{\frac{2m^{*}k_{B}T}{\hbar^{2}}}{ℑ}_{-1/2}\left(\frac{E_{F}-E_{k}}{k_{B}T}\right)\text{,}$$

where *k*_B_ is Boltzmann's constant, *T* is the temperature, *E*_F_ is the Fermi level, *E*_*k*_ the energy of the bottom of the *k*th sub-band, and *ℑ*_−1/2_ the Fermi-Dirac integral of order −1/2. The factor in front of *ℑ*_−1/2_ comes from the 1D density of states (DOS), and the units of *n*_k_ are those of a line density (*m*^−1^), which when multiplied by the normalized probability density gives the volume density *n*_1DEG_(*r*). The overall charge density *ρ*(*r*) is then given by the following:

(7) $$\rho \left(r\right)=e\left({N_{D}}^{+}\left(r\right)-n_{1\mathrm{DEG}}\left(r\right)\right)$$

where *e* is the electron charge and *N*_D_^+^(*r*) is the distribution of the ionized donor impurities. Using *ρ*(*r*) and *V*(*r*) which is related to the electrostatic potential *ϕ*(*r*) via *V*(*r*) = −*e*ϕ(*r*), it is possible to solve the Poisson equation in cylindrical coordinates:

(8) $$\frac{\partial^{2}\varphi }{\partial r^{2}}+\frac{1}{r}\frac{\partial \varphi }{\partial r}=-\frac{\rho \left(r\right)}{\epsilon_{o}\epsilon_{r}}$$

where *ϵ*_o_ is the permittivity of free space and *ϵ*_r_ is the relative permittivity. The exact or self-consistent potential *ϕ*_0_(*r*) is expressed in terms of the trial potential *ϕ*(*r*) and a correction potential *δϕ*(*r*)

(9) $$\varphi_{o}\left(r\right)=\varphi \left(r\right)+\delta \varphi \left(r\right)\text{.}$$

This is substituted into Poisson's equation which is solved to find *δϕ*(*r*). However, it is necessary to find an expression for the change in the quantum density *n*_1DEG_(*r*) given a small change *δϕ*. This is required since *n*_1DEG_(*r*) is also dependent on the potential *ϕ*, i.e., *n*_1DEG_(*r*,*ϕ*). A perturbation *ϕ* → *ϕ* + *δϕ* will change the quantum electron density from *n*_1DEG_(*ϕ*) to *n*_1DEG_(*ϕ* + *δϕ*)

(10) $$n_{1\mathrm{DEG}}\left(\varphi +\delta \varphi \right)=n_{1\mathrm{DEG}}\left(\varphi \right)+\delta n_{1\mathrm{DEG}}\left(\varphi ,\delta \varphi \right)\text{.}$$

The approach of Trellakis et al. [7] who derives an expression for *δn*_1DEG_(*ϕ*,*δϕ*) applicable to quantization in an infinite nanowire of square cross-section is adopted, and then using the derivative property of the Fermi-Dirac integrals simplifies *n*_1DEG_(*ϕ* + *δϕ*) to

(11) $$n_{1\mathrm{DEG}}\left(\varphi +\delta \varphi \right)=\frac{1}{\pi }\sqrt{\frac{2m^{*}k_{B}T}{\hbar^{2}}}\sum_{k}{\psi_{k}}^{2}\left(\varphi \right){ℑ}_{-3/2}\left(\frac{E_{F}-E_{k}+\mathrm{q\delta \varphi}}{k_{B}T}\right)\text{.}$$

Using *δn*_1DEG_(*ϕ*,*δϕ*)and *n*_1DEG_(*ϕ* + *δϕ*) in Poisson's equation, yields an expression that may be expressed in matrix form and is effectively a linear system of equations in the unknown vector *δϕ*. This has been described in detail elsewhere [3]. After adding *δϕ*(*r*,*z*) onto the trial potential *ϕ*(*r*,*z*), the process of solving the Poisson-Schrödinger equations is repeated until convergence is reached, i.e., when the average of the *δϕ*(*r*,*z*) is typically <0.1 meV and charge neutrality is achieved and checked for completeness.

## Results and discussion

In the case of InAs/InP/InAs/InP/InAs core-shell NW RTDs, the InAs and InP shells are lattice matched, and we take into account (a) the position of the Fermi level with respect to the CB edge at the surface to be pinned or fixed ≈ 100 meV below the Fermi level; (b) the dielectric permittivity *ϵ*_R_ = 15.1 and effective mass of electrons *m*_e_^*^ = 0.023 m_o_ in InAs; (c) *ϵ*_R_ = 13.1 and *m*_e_^*^ = 0.08 m_o_ in InP; and (d) the CB discontinuity at the InAs/InP interface, i.e., Δ*E*_C_ ≈ 0.6 eV €, and a background doping level of *N*_D_ = 1 × 10^16^ cm^−3^[3]. In the case of the GaAs/Al_0.3_Ga_0.7_As/GaAs/Al_0.3_Ga_0.7_As/GaAs core-shell NW RTDs, GaAs and Al_0.3_Ga_0.7_As are also lattice matched and the GaAs/Al_0.3_Ga_0.7_As heterojunction of type I [8,9]. In addition, we take into account (a) the Fermi level to be pinned at the middle of the gap, i.e., 0.7 eV below the conduction band edge at the surface; (b) the dielectric permittivity *ϵ*_R_ = 13.1 and effective mass of electrons *m*_e_^*^ = 0.067 m_o_ in GaAs; (c) *ϵ*_R_ = 13.1 and *m*_e_^*^ = 0.092 m_o_ in Al_0.3_Ga_0.7_As; and (d) the CB discontinuity at the GaAs/Al_0.25_Ga_0.75_As interface, i.e., Δ*E*_C_ ≈ 0.6 Δ*E*_G_ = 0.25 eV.We consider first the case of the InAs/InP/InAs/InP/InAs core-shell NW RTD in equilibrium in order to obtain an understanding of the electronic sub-band structure and how this depends on the thickness of the various shells. A schematic diagram of the InAs/InP/InAs/InP/InAs core-shell NW RTD is shown as an inset in Figure 1a,b,c.

The potential profile, energetic position of the 1DEG sub-bands, and corresponding wave functions in a 10 nm InAs/10 nm InP/8 nm InAs/10 nm InP/10 nm InAs core-shell NW RTD are shown in Figure 1a. The wave functions shown for *n* =1 and *n* = 2 are normalized and offset for clarity only in the vertical direction. As stated above, we assume that the conduction band potential is circularly symmetric, so only half of the potential profile is shown beginning at the core where *r* = 0 and ending at the surface where *r* = 50 nm. In addition, the conduction band potential profile is shown versus distance, i.e., *E*_C_(*r*) with respect to the Fermi level, *E*_C_(*r*) − *E*_F_, where *E*_F_ = 0 eV. The energetic position of the Fermi level with respect to the conduction band edge is known and fixed, i.e., pinned at the surface so the energies of the 1DEG sub-bands, i.e., *E*_*n*,*m*_ in the various segments are also given with respect to *E*_F_ which is a convention also used previously [3].

Now, the potential profile of the 10 nm InAs/10 nm InP/8 nm InAs/10 nm InP/10 nm InAs core-shell NW RTD shown in Figure 1a resembles a top-hat double barrier, and there is little band bending while the lowest sub-band in the 8 nm InAs middle shell belonging to the *m* = 0 ladder of states is 72 meV above the Fermi level. We also find a second sub-band in the InAs middle shell close to the top of the barrier, i.e., 414 meV above the Fermi level. On the other hand, the lowest sub-band belonging to the *m* = 0 ladder of states in the core resides just 6 meV above the Fermi level while that in the surface shell falls 10 meV below the Fermi level. Evidently, the lowest sub-band in the InAs middle shell is energetically higher than those in the core and shell as required for a RTD operation.

For completeness, the electronic sub-band structure of the *m* = 1 ladder of states is shown in Figure 1b. Both *m* = 1 sub-bands in the InAs middle shell are energetically close to those with *m* = 0 and have more or less identical wave functions. However, the lowest sub-band in the core with *m* = 1 has a node at *r* = 0 but resides 103 meV above the Fermi level which is considerably higher than 6 meV for *m* = 0 so it is not expected to contribute significantly towards the current transport.

Here, it should be noted that the application of an external electric field or applied voltage between the core and surface shell as shown by the inset in Figure 1b, i.e., positive surface and negative core potential, will make the sub-bands in the InAs middle shell fall in energy and line up with the quasi Fermi level in the core allowing carriers to tunnel from the core into the surface shell through the double barriers as shown by the inset in Figure 1c. Hence, the voltage at which the onset of tunneling occurs may be adjusted via the energetic position of the lowest sub-band in the InAs middle shell by changing its thickness. The dependence of the *E*_(1,0)_ sub-band in the InAs middle shell versus its thickness is shown in Figure 1c, and the onset of resonant tunneling will occur at larger voltages upon decreasing the thickness of the middle shell and conversely smaller voltages for larger thicknesses. However, the latter must not exceed a critical thickness since it will inadvertently lead to a deterioration of device performance due to the formation of defects.We find that the lowest sub-band energy in the middle shell changes from 25 meV up to 480 meV above the Fermi level by decreasing its thickness from 10 nm down to 1 nm as shown in Figure 1c. Consequently, the optimum middle shell thickness must not exceed 10 nm in order to maintain the lowest sub-band in the middle shell above the Fermi level.

It should also be noted that the wave functions in the middle shell do not decay towards zero in the barriers having thicknesses of <5 nm but extend into the core and surface shells which is not desirable for optimum performance. One might suppress the penetration of the wave functions by increasing the barrier thickness, but this will inadvertently result into a reduction of the transmission probability and current which in turn will increase the overall resistance of the device. We find that the confinement of the wave functions and sub-band energies in the middle shell changes slightly by increasing the barrier thickness from 5 to 10 nm, hence the optimum thickness of the barriers is ≈ 5 nm similar to the thickness of the InP barriers in the axial InAs/InP/InAs/InP/InAs NW RTD of Björk et al. [1].

We have shown that the lowest *m* = 0 and *m* = 1 sub-bands in the middle shell of the InAs/InP/InAs/InP/InAs core-shell NW RTD shown in Figure 1a,b reside energetically above the two sub-bands in the core and surface shell thickness. However, the lowest sub-band belonging to the *m* = 0 ladder of states in the core resides 6 meV above the Fermi level while that in the surface shell falls 10 meV below the Fermi level. Therefore, it is necessary to increase the core radius so that a single sub-band falls below the Fermi level in the core which will act as an emitter providing electrons for resonant tunneling. Here, it should be noted that the energetic position of the lowest sub-band in the core with respect to the Fermi level dictates the full-width half-maximum (FWHM) of the current–voltage peak so it is necessary to find how this varies by changing the core radius.

**Figure 2:**
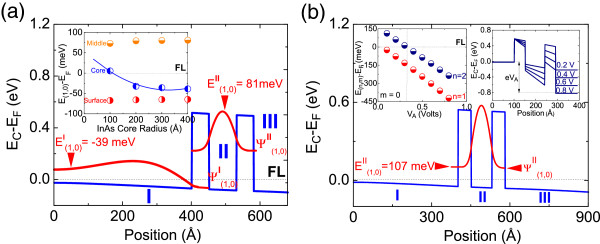
**CB potential profile, sub-band energies, and wave functions. (a)** in a 40 nm InAs/5 nm InP/8 nm InAs/5 nm InP/10 nm InAs core-shell NW RTD; inset shows variation of the sub-band energies in the core, middle, and surface shells versus core radius. **(b)** Same as (a) but with a 40-nm InAs surface shell; insets show the variation of the sub-bands in a 10-nm InAs middle shell with *V*_A_ and the potential profile for *V*_A_ = 0.2, 0.4, 0.6, and 0.8 V.

The SCPS potential profile, sub-band energies and wave functions of a 40 nm InAs/5 nm InP/8 nm InAs/5 nm InP/10 nm InAs core-shell NW RTD is shown in Figure 2a while the variation of the lowest sub-band energy in the core, middle, and surface shells for different core radii is shown as an inset in Figure 2a. The lowest sub-band in the 8 nm middle shell of Figure 2a resides 80 meV above the Fermi level and that in the 20 nm InAs surface shell 65 meV below the Fermi level, but these are not strongly dependent on the core radius as shown by the inset of Figure 2a. In contrast, the lowest *m* = 0 sub-band in the core is energetically located 5 meV above the Fermi level and falls to 40 meV below the Fermi level upon increasing the core radius from 10 to 40 nm. We also observe the emergence of a second sub-band ≈ 13 meV below the Fermi level for a core radius of 40 nm. Interestingly, the wave function of the lowest *m* = 0 sub-band in the core has a maximum closer to the barriers, as shown in Figure 2a, rather than *r* = 0 as can be seen in Figure 1a but *∂ψ*/*∂r* = 0 still holds at *r* = 0. The case of a symmetric 40 nm InAs/5 nm InP/8 nm InAs/5 nm InP/40 nm InAs core-shell NW RTD is also shown for comparison in Figure 2b. The potential exhibits a linear variation with distance, but now there are two sub-bands below the Fermi level in the surface shell and only one in the core while the lowest sub-band in the middle shell has changed from 81 to 107 meV due to an increase in the overall band bending and potential profile along the radius.

So far, we have considered the InAs/InP/InAs/InP/InAs core-shell NW RTD in equilibrium and determined that an optimum configuration is obtained for core radii and surface shell thicknesses >10 nm, middle shells <10 nm, and 5 nm InP barriers.The application of an external electric field requires the fabrication of separate ohmic contacts to the core and surface shell as shown in Figure 1b and extra processing compared to the case of axial NW RTDs. Electrons tunnel, one at a time, via discrete states confined in the middle InAs cylindrical quantum dot in axial InAs/InP/InAs/InP/InAs NW RTDs. In contrast, many electrons can tunnel at the same time via one-dimensional sub-band states confined in the middle shell of a InAs/InP/InAs/InP/InAs core-shell NW RTD giving rise to a larger current-carrying capability.The application of a potential difference as shown in the inset of Figure 1b will make the lowest sub-band in the InAs middle shell fall towards the quasi Fermi level in the core, causing an increase in current, that will flow from the core into the surface shell corresponding to resonant tunneling via the double barriers. This is shown by the inset in Figure 1c. The current will reach a maximum and will decrease back to zero when the sub-band in the middle shell falls below the sub-band confined in the core as the electric field or applied voltage is increased further.

The potential profile of the 10 nm InAs/5 nm InP/8 nm InAs/5 nm InP/10 nm InAs core-shell NW RTD under the application of an external electric field and different applied voltages is also shown as an inset in Figure 2b where the potential difference is taken to appear across the intrinsic-like region defined by the InP barriers and InAs middle shell. In other words, the core and surface shells are metallic-like and have a small resistance similar to the case of axial NW RTDs [3,4]. The variation of the *m* = 0, *n* = 1, and *n* = 2 sub-bands *E*_(*n*,*m*)_ in the 8 nm InAs middle shell of the 10 nm InAs/5 nm InP/8 nm InAs/5 nm InP/10 nm InAs core-shell NW RTD with applied voltage is also shown as an inset in Figure 2b. Both *E*_*nm*_, i.e., *E*_(1,0)_ and *E*_(2,0)_ in the middle shell vary linearly with applied voltage and *δE*_*nm*_/*δV*_A_ ≈ 560 meV/V for both sub-bands.

In particular, we find that the *n* = 1 sub-band in the middle shell coincides with the quasi Fermi level at *V*_A_ ≈ 50 mV which will give rise to the first current–voltage peak after which the *n* = 2 sub-band will coincide with the Fermi level at *V*_A_ ≈ 325 mV leading again to an increase of the current and a second peak. It is worthwhile pointing out that the current is expected to exhibit a continuous increase for *V*_A_ > 0.8 V when the top of the right barrier falls below the quasi Fermi level in the core as shown in the inset of Figure 2b in which case carriers will essentially tunnel through a single barrier from the core into the surface.

Reversing the polarity of the applied voltage or electric field will make the lowest sub-band in the middle shell fall towards the quasi Fermi level on the surface side, causing an increase in current, that will flow from the surface into the core. However, the double-barrier potential profile shown in Figure 2a,b is linear but not top-hat or flat-band-like as shown in Figure 1a,b so the core-shell NW RTD of Figure 2a,b will exhibit an asymmetry in the current–voltage characteristics. In other words, the application of an external electric field will lead to the onset of resonant tunneling at slightly different applied voltages depending on the polarity. However, a detailed description of the transmission probability versus applied voltage and current transport through both *m* = 0 and *m* = 1 ladder of states in such core-shell NW RTDs will be given in detail elsewhere.

**Figure 3:**
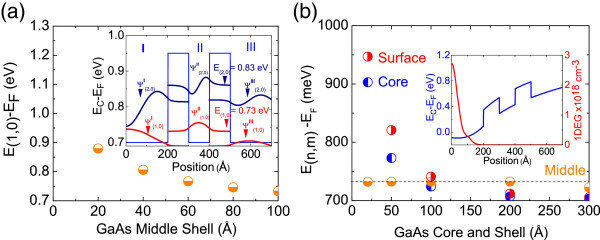
**Variation of the lowest sub-band energy. (a)** In the middle shell of 20 nm GaAs/10 nm Al_0.3_Ga_0.7_As/10 nm GaAs/10 nm Al_0.3_Ga_0.7_As/20 nm GaAs core-shell NW. **(b)** In the core, middle, and surface shells versus thickness of core taken to be equal to the surface shell.

For completeness, we consider also the case of GaAs/AlGaAs/GaAs/AlGaAs/GaAs core-shell NW RTDs. The potential profile of an all-intrinsic 20 nm GaAs/10 nm AlAs/10 nm GaAs/10 nm AlAs/20 nm GaAs core-shell NW RTD is shown as an inset in Figure 3a. The top-hat CB potential profile is flat since there are no ionized impurities, and the Fermi level is pinned at the middle of the energy gap, i.e., 0.7 eV below the conduction band edge at the surface, i.e., *E*_C_ − *E*_F_ = 0.7 eV. There are two bound states in the 10 nm GaAs middle shell at 33 and 126 meV above the CB edge or 733 and 826 meV above the Fermi level which belong to the *m* = 0 ladder of states and their corresponding wave functions decay to zero in the barriers. We find only one sub-band in the middle shell at 32 meV above the CB edge while the wave functions and energies of the *m* = 0 and *m* = 1 sub-bands in the middle shell are similar.

The variation of the lowest sub-band energy in the middle shell versus its thickness is shown in Figure 3a and increases from 33 meV up to 200 meV above the CB edge upon reducing the thickness of the middle shell from 10 to 2 nm. This is different to the case of InAs shown in Figure 1b where the lowest *m* = 0 sub-band changed from 172 meV up to 500 meV above the CB edge upon reducing the thickness of the middle shell from 10 to 2 nm and is a direct consequence of the lower effective mass of electrons in InAs compared to GaAs. Consequently, smaller turn on voltages or applied electric fields is required in the case of GaAs core-shell NW RTDs having equal shell thicknesses with InAs.

The variation of the lowest *m* = 0, *n* = 1 sub-band energies in the core, middle, and surface shells of the same core-shell NW RTD versus core radius is shown in Figure 3b. For core radii <10 nm, the lowest sub-band in the core and surface shells resides energetically above that in the middle shell. We find that the core radius and surface shell thickness must be >20 nm in order to obtain the desired energy configuration and sub-band line up.

The GaAs/AlGaAs/GaAs/AlGaAs/GaAs core-shell NW RTDs considered in Figure 3a,b were taken to be intrinsic in order to show how the electronic sub-band structure depends on the core radius and shell thickness. Doping is required to obtain carriers for device operation and the SCPS potential profile of a modulation doped GaAs/AlGaAs/GaAs/AlGaAs/GaAs core-shell NW RTD with *N*_D_ = 3 × 10^18^ cm^−3^ donor impurities in the core is shown as an inset in Figure 3b along with the 1DEG charge distribution which has a maximum at *r* = 0. The potential has a U-like shape across its diameter and two sub-bands fall at 13 and 73 meV below the Fermi level in the core while the lowest sub-band in the middle shell resides 382 meV above the Fermi level, but the surface shell is depleted. The application of a negative surface potential would result into its complete depletion and no current flow. In contrast, a current will flow from the core into the surface shell by applying a positive surface potential. From above, it is clear that InAs/InP/InAs/InP/InAs core-shell NW RTDs are more attractive not only since they have a symmetric, top-hat potential profile but also in view of the fact that it is easier to make ohmic contacts on InAs than GaAs core-shell NW RTDs which require doping. Finally, it should be noted that the growth and fabrication of GaN core-shell NW RTDs [10] is more difficult compared to the case of GaAs or InAs core-shell NW RTDs especially as nitrides have an inherently higher density of crystallographic defects and interface charges that may give rise to asymmetric potential profiles.

## Conclusions

The electronic properties of InAs/InP/InAs/InP/InAs core-shell NW RTDs such as the potential profile, energetic position of the one-dimensional sub-bands, and corresponding wave functions have been investigated via the self-consistent solution of the Poisson-Schrödinger equations in the effective mass approximation taking into account a one-dimensional density of states by varying the core radius and different shell thicknesses. An optimal energy configuration is obtained for core radii >10 nm, middle shells <10 nm, and 5 nm InP barriers. The sub-bands belonging to the *m* = 0 and *m* = 1 ladder of states in the middle shell have similar wave functions and energies. In contrast, the *m* = 1 states in the core are not expected to play a significant role in the current transport since they reside energetically well above the Fermi level. Smaller turn on voltages may be achieved in the case of GaAs/AlAs/GaAs/AlAs/GaAs core-shell NW RTDs due to the larger effective mass, but doping is required so that carriers are available for resonant tunneling. Such core-shell NW RTDs have a higher current-carrying capability compared to their axial counterparts and are expected to be important for the fabrication of high speed nanoelectronic devices and energy conversion applications.

## Competing interests

The author declares that he has no competing interests.
